# Macrophages: a double-edged sword in female reproduction and disorders

**DOI:** 10.1038/s12276-025-01392-6

**Published:** 2025-02-03

**Authors:** Mira Park, Yeon Sun Kim, Haengseok Song

**Affiliations:** 1 Department of Biomedical Science, College of Life Science, CHA University, Pocheon, Korea; 2 Division of Life Science, CHA University, Pocheon, Korea; 3 Department of Life Science, Graduate School, CHA University, Pocheon, Korea; 4 CHA Advanced Research Institute, Seongnam, Korea; 5 KW-Bio Co., Chuncheon, Korea

**Keywords:** Reproductive disorders, Monocytes and macrophages

## Abstract

Reproduction consists of sequential inflammation-like events, primarily within the endometrium, from ovulation to embryo implantation, decidualization and delivery. During the reproductive cycle, the endometrium repeatedly undergoes cyclic periods of proliferation, differentiation, tissue breakdown and repair without scarring. Owing to their phagocytic activity, macrophages, key players in innate immunity, are thought to play crucial roles in the endometrium. Endometrial macrophages actively participate in various stages of reproductive tissue remodeling, particularly during decidualization and pregnancy establishment. Traditionally considered simple bystanders that clear debris to prevent autoimmune responses in tissue homeostasis, macrophages are now recognized as main actors with broad functional plasticity that allows them to fine tune the balance between pro- and anti-inflammatory responses during tissue inflammation, remodeling and repair. Homeostatic balance is determined by the sum of various mediators produced by two distinctly polarized macrophage subpopulations. The biased polarization of tissue-resident macrophages may contribute to the pathogenesis of various diseases, such as inflammation and cancer. Thus, understanding how macrophages contribute to endometrial homeostasis is crucial for deciphering the underlying mechanisms of various reproductive disorders. Nanomedicines using extracellular vesicles, nanoparticles and noncoding RNAs have recently been applied to modulate macrophage polarization and alleviate disease phenotypes. Despite these advances, the functions of endometrial macrophages under physiological and pathophysiological conditions remain poorly understood, which complicates the development of targeted therapies. Here we update the current understanding of the homeostatic function of macrophages and the putative contribution of endometrial macrophage dysfunction to reproductive disorders in women, along with innovative molecular therapeutics to resolve this issue.

## Understanding macrophage roles in reproductive tissue dynamics

Macrophages are a type of white blood cell that plays a key role in our immune system by engulfing and digesting harmful substances. This Review explores the diverse roles of macrophages beyond their basic immune functions, particularly in female reproduction. Researchers found that these cells are crucial for inflammation regulation and tissue repair. Macrophages can change their behavior based on signals from their environment, which is known as polarization. They can become either M1 or M2 types, depending on the situation. In female reproduction, macrophages help with processes such as menstruation, embryo implantation and pregnancy maintenance. The study concludes that understanding macrophage functions can lead to new treatments for reproductive issues. Future research may focus on using macrophage-targeted therapies to improve reproductive health and treat related diseases.

This summary was initially drafted using artificial intelligence, then revised and fact-checked by the author.

## Introduction

Macrophages (meaning ‘large eaters’ in Greek) are a type of white blood cell in the innate immune system that patrols by ameboid movement to find potential pathogens, such as microbes, cellular debris and foreign substances, which they engulf and digest via phagocytosis. Macrophages are found in all species, from invertebrates to mammals, and play crucial roles in evolution through their ability to perform phagocytosis. In vertebrates, macrophages are found in nearly every tissue from the early stages of development and persist throughout the lifetime of the organism. In addition, cells of the myeloid lineage are recruited to the site of injury or disease, even in the absence of pathogens, and are dispersed after repair or recovery^1^. These behaviors indicate that macrophages are more than simple bystanders that perform unsophisticated phagocytic functions. Tissue-resident macrophages are among the first immune cells to respond to tissue damage, producing almost all known effectors that initiate and orchestrate the recruitment of granulocytes from the blood into tissue^2^. The coordinated release of these factors enables macrophages to dramatically affect their environment.

Macrophages play critical roles both in tissue remodeling under normal physiological conditions and in resolving inflammation and tissue injury^3^. Macrophages are highly capable of efferocytosis, which facilitates the clearance of dead cells, thereby suppressing tissue necrosis and promoting resolution and repair^4^. During tissue injury, substantial numbers of neutrophils infiltrate the affected areas. Most neutrophils rapidly undergo apoptosis and begin to accumulate as uncleared apoptotic cells, and then macrophage-mediated efferocytosis and neutrophil clearance are performed during the resolution stage. These observations support the idea that efferocytosis of macrophages by apoptotic neutrophils is a key immune trigger for the onset of resolution^5^. In addition to phagocytosis, a nonspecific defense mechanism in innate immunity, macrophages serve as antigen presenters for T cells to promote specific defense mechanisms in adaptive immunity. The multifaceted functionality of macrophages positions them as pivotal orchestrators capable of exerting nuanced effects not only on immune responses but also on developmental processes and tissue homeostasis^6^. Here, we focus on the major functions of macrophages beyond their role in innate immunity: macrophages contribute to tissue homeostasis, remodeling and repair in the endometrium for successful reproduction.

## Tissue-resident macrophages

**Fig. 1:**
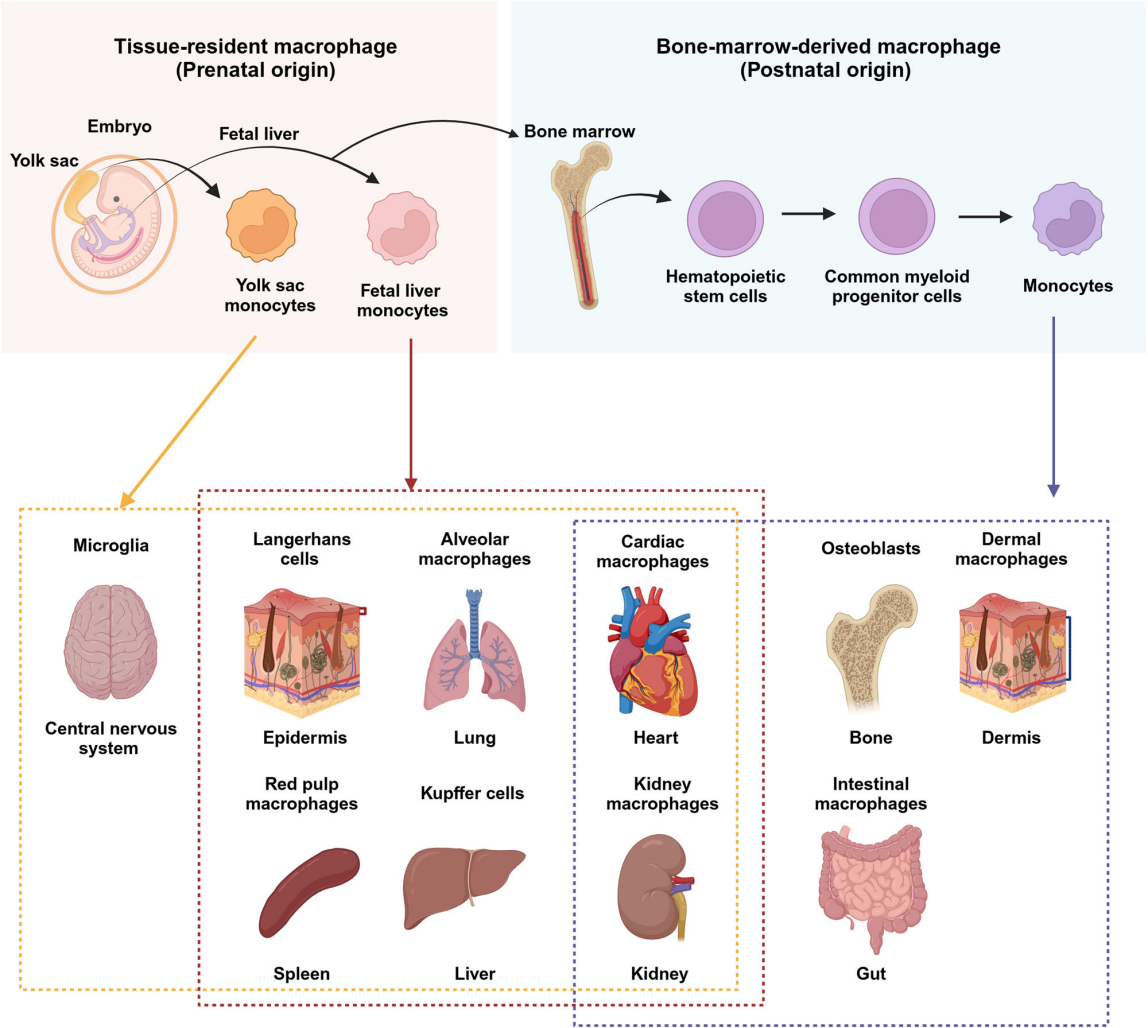
Ontogeny and distribution of tissue-resident macrophages. Origin and distribution of tissue-resident macrophages in various organs of the body. Each macrophage subtype performs specific roles in tissue homeostasis, inflammation and immune responses on the basis of its local environment. The image was created with BioRender.com.

Elie Metchnikoff, a Russian zoologist, first identified macrophages in 1882 when he observed interesting cells that encircled and devoured a rose thorn introduced into a transparent starfish larva. To determine the irreplaceable roles of these cells in host defense and the phagocytosis of unwanted cells during development, injury and repair^6^, Elie Metchnikoff received the Nobel Prize for Physiology or Medicine in the year 1908. Macrophages are now known as the main regulators of inflammation and subsequent repair. Resident macrophages in healthy adult tissues are either derived from circulating monocytes or established before birth and are maintained independently of monocytes during adult life. These cells together form the ‘mononuclear phagocyte system’, and each type of macrophage is designated by a unique name based on its location within the body (Fig. 1)^7^. In contrast, most macrophages that accumulate in diseased or damaged tissues are derived from circulating monocytes. Monocytes are attracted to unhealthy areas by chemotaxis, which is triggered by a range of stimuli, including damaged cells, pathogens and cytokines released by macrophages already on site. Unlike short-lived neutrophils, macrophages survive in the body for several months.

Resident macrophages in adult tissues can be developmentally derived from three sources. The first is the extraembryonic yolk sac in the embryo during early gestation (embryonic days 6.5 (E6.5)–E8.5) in mice. At this stage, macrophages are the only ‘white blood cells’ because primitive progenitor cells give rise only to macrophages and red blood cells. The second source is definitive hematopoietic stem cells (HSCs), which contribute to all immune lineages at E8.5–E10.5. These HSCs then migrate to the fetal liver, which serves as the primary hematopoietic organ during the remainder of fetal development. The third source is the bone marrow (BM), where definitive hematopoiesis occurs in both fetuses and adults^7^. Subtypes of myeloid cell have heterogeneous developmental origins. In the central nervous system, microglia derived from the embryonic yolk sac are not replenished by blood-derived infiltrating monocytes. In contrast, resident macrophages in other tissues derived from HSCs in the BM can be expanded either by local proliferation in situ or by infiltration, depending on the stimuli^8^. Although macrophages and other immune cells continue to migrate in and out of the endometrium during the reproductive cycle, the composition of endometrial macrophages remains unclear.

## Phenotype and function of polarized macrophages

**Fig. 2:**
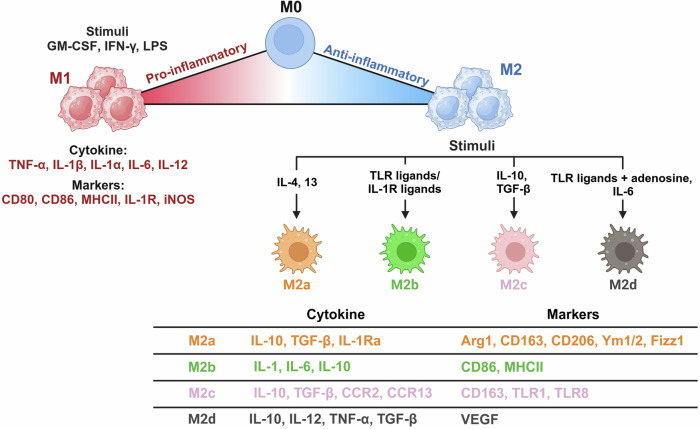
Macrophage polarization states and their functionalities. Polarization states of macrophages (M1 and M2) with their stimuli, markers and secreted cytokine profiles. Macrophages can adopt a spectrum of activation states in response to various signals, resulting in distinct functional phenotypes. The image was created with BioRender.com.

The phenotypes and functions of tissue-resident macrophages depend mainly on local environmental cues that induce unique endogenous profiles. Epigenetic landscapes, transcription factors and noncoding RNA networks underlie adaptability to a dynamic spectrum of macrophage phenotypes that can be activated or suppressed by the local niche. Macrophages are grouped into proinflammatory (M1) and anti-inflammatory/prorepair (M2) phenotypes on the basis of the expression of key surface markers and the production of certain cytokines^3^. However, it is widely accepted that the M1/M2 dichotomous paradigm of macrophage polarization is oversimplified. It does not accurately account for macrophage phenotypes in different contexts. Currently, there is still no standardized nomenclature that can fully cover the diversity of macrophage origins, stimuli and microenvironments. Although M1- and M2-polarized macrophages are extremes of a continuum of activation states, we define macrophage polarity with an M1/M2 dichotomy in this review to understand the complex plasticity of macrophages (Fig. 2).

When activated by various stimuli, such as interferon-γ (IFN-γ), granulocyte‒macrophage colony‒stimulating factor (GM-CSF) and microbial components, including lipopolysaccharide (LPS), macrophages are polarized to the M1 phenotype, also known as classically activated macrophages. M1 macrophages participate in the Th1 response to protect the host against viruses and intracellular bacteria during acute infections or tumors^9^. They produce proinflammatory factors, including tumor necrosis factor (TNF) and interleukins (ILs), such as IL-1, IL-6, IL-12 and IL-23. Macrophages can sense danger signals through pattern recognition receptors such as Toll-like receptors (TLRs), which recognize bacterial moieties. Specifically, LPS and other microbial ligands stimulate TLR4, activating pathways to promote the expression of various proinflammatory cytokines (including TNF, IL-1β and IL-12), chemokines (CXC motif chemokine ligand 10 and 11) and costimulating proteins^6^.

When activated by IL-4 or IL-13, macrophages are instead polarized to the M2 phenotype. M2 macrophages contribute to the development of an anti-inflammatory milieu that allows the resolution of inflammation, followed by tissue repair. These cells express high levels of soluble IL-1 receptor (sIL-1R) as a decoy receptor, an IL-1R antagonist with anti-inflammatory properties and a mannose receptor (CD206) to scavenge unwanted mannoglycoproteins for immune homeostasis. M2 macrophages also produce profibrotic factors, such as transforming growth factor β (TGF-β) and insulin-like growth factor 1 (IGF-1), thus actively suppressing inflammation and promoting repair^10^. The common signatures of this subpopulation are IL-10^high^IL-12^low^ and arginase 1 (ARG1) expression. Increased ARG1 depletes extracellular l-arginine, leading to impaired T cell proliferation and IFN-γ production. ARG1 further competes with inducible nitric oxide synthase (iNOS) for l-arginine and reduces nitric oxide (NO) production^11^. Increased arginase activity results in the production of polyamines and collagen, which promote tissue remodeling and wound healing. M2 macrophages induce angiogenesis and lymphangiogenesis by producing vascular endothelial growth factor A (VEGF-A), epidermal growth factor, platelet-derived growth factor and IL-8 (ref. ^12^). Markers and/or effectors associated with M2 polarization include signal transducer and activator of transcription 6, suppressor of cytokine signaling 1, peroxisome proliferator-activated receptor gamma (PPARγ), CD163, CD36 and matrix metalloproteinases 9 (ref. ^11^).

M2 macrophages are further divided into M2a, M2b, M2c and M2d subtypes on the basis of the applied stimuli and their transcriptional profiles. M2a, which is related to the Th2 immune response, is stimulated by IL-4, IL-13 or fungal and helminth infections. CD86 is a marker for discriminating M2b macrophages from other M2 subtypes, but it is also found in M1 macrophages. M2c is induced by IL-10 and TGF-β and contributes to tissue remodeling and extracellular matrix production. M2d polarization is activated by IL-6, TLR ligands and adenosine^13,14^. Macrophages within tumors, termed tumor-associated macrophages (TAMs), exhibit functional features similar to those of M2 macrophages and can be characterized as M2d. During tumor development, tumor-infiltrating M1 macrophages with an IL-10^low^IL-12^high^ phenotype initially promote immune responses, instigating tumor disruption. However, TAMs generally switch to an IL-10^high^IL-12^low^ M2-like phenotype with low tumoricidal activity during the late stages of tumorigenesis. These TAMs facilitate angiogenesis to provide a favorable microenvironment for tumor growth and survival. Although TAMs exhibit an anti-inflammatory phenotype, activated TAMs can produce proinflammatory cytokines, such as IL-6, to promote tumor cell cycle progression and suppress apoptosis^15^. The dual nature of TAMs, with both anti-inflammatory and proinflammatory attributes, underscores their versatile and context-dependent behavior in tumorigenesis.

## Metabolic regulation of macrophage polarization

**Fig. 3:**
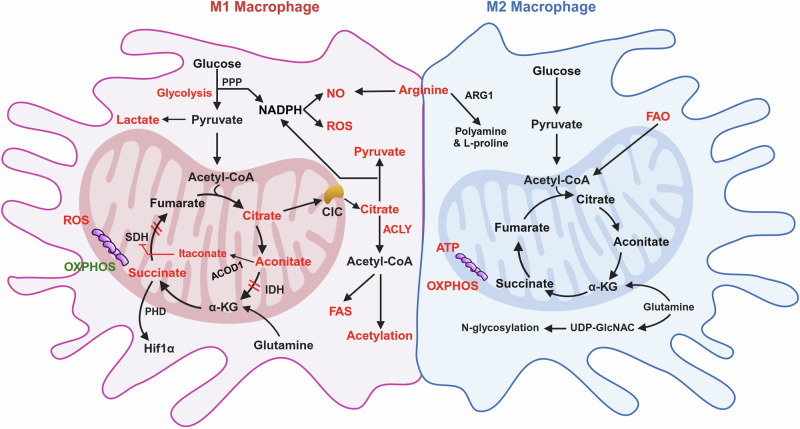
Metabolic pathways in M1 and M2 macrophages. Distinct metabolic pathways utilized by M1 and M2 macrophages highlight their differential roles in immune responses. M1 macrophages rely primarily on glycolysis and the PPP and produce ROS and NO to support their proinflammatory function. On the other hand, M2 macrophages depend on OXPHOS and FAO, which are associated with tissue repair and anti-inflammatory roles. The image was created with BioRender.com.

The differential polarization of macrophages in response to microenvironmental cues can trigger distinct changes in their metabolic programs. Macrophages use major metabolic pathways and the mitochondrial tricarboxylic acid (TCA) cycle to fine tune their plasticity in a context-dependent manner (Fig. 3). Proinflammatory M1 macrophages utilize glycolysis and the pentose phosphate pathway (PPP) to meet ATP requirements because the TCA cycle is disrupted at two points, and oxidative phosphorylation (OXPHOS) and fatty acid oxidation (FAO) are downregulated. In contrast, the metabolic activity of M2 macrophages is characterized by increased OXPHOS and FAO in the intact TCA cycle^12^.

### Metabolic changes in M1 macrophages

Glycolysis is a crucial metabolic event in M1 macrophages, and its inhibition affects many major inflammatory phenotypes, including phagocytosis, reactive oxygen species (ROS) production and the secretion of proinflammatory cytokines. Glycolysis converts glucose to pyruvate in the cytosol, generating two ATPs per glucose. It also supplies the PPP, allowing the production of nicotinamide adenine dinucleotide phosphate (NADPH). NADPH oxidases catalyze NADPH to produce ROS, which are essential for the phagocytic activity of M1 macrophages. In M1 macrophages, the TCA cycle is interrupted at two key points, allowing signaling metabolites (citrate, succinate and itaconate) to escape the mitochondria or perform their regulatory role in macrophage polarity^12^.

Citrate is converted to isocitrate and then to α-ketoglutarate (α-KG) by isocitrate dehydrogenase (IDH) in the TCA cycle. Citrate accumulates in M1 macrophages owing to two main transcriptional alterations: IDH downregulation and mitochondrial citrate carrier (CIC) upregulation. Both events are responsible for the initial interruption of the TCA cycle and consequent citrate accumulation in the cytosol of M1 macrophages^12^. CIC is responsible for the efflux of acetyl-CoA from mitochondria to the cytosol in the form of citrate. These typical features of M1 macrophages allow citrate to flow out of the mitochondria. ATP citrate lyase (ACLY) cleaves citrate in the cytosol to produce acetyl-CoA for de novo lipogenesis. ACLY shuttles between the cytosol and nucleus, where it generates acetyl-CoA for histone acetylation^16^. Excess citrate is also used to synthesize acetyl-CoA, which supports fatty acid synthesis to meet the demands of inflammatory macrophages. Blocking the acetyl-CoA supply by downregulating CIC can lead to defective macrophage responses to LPS.

Itaconate is synthesized through the decarboxylation of citrate-derived aconitate by aconitate decarboxylase 1 in the mitochondrial matrix. Endogenous itaconate inhibits succinate dehydrogenase (SDH), which mediates succinate oxidation, leading to succinate accumulation in macrophages exposed to LPS. This is closely associated with reduced mitochondrial respiration, ROS production, proinflammatory cytokine secretion and inflammasome activation. Accordingly, reduced itaconate levels may favor M2-like polarization because of the release of the SDH brake, which improves OXPHOS flux^12^. The increase in succinate because of the release of the SDH brake could stabilize hypoxia-induced factor 1 alpha (HIF1α) by inhibiting prolyl hydroxylases (PHDs), which typically promote HIF1α degradation in an oxygen-dependent manner. By inhibiting PHDs, succinate prevents HIF1α degradation even in the presence of oxygen, thus blocking HIF1α degradation even in the presence of oxygen and enabling the continued transcription of genes associated with hypoxia and metabolic reprogramming.

### Metabolic changes in M2 macrophages

In contrast to glycolytic M1 macrophages, M2 macrophages rely mainly on the TCA cycle and OXPHOS for glucose metabolism and energy generation. This metabolic pathway allows efficient and sustained energy generation, a prerequisite for reparative M2 macrophages to support tissue repair^12^. The inhibition of iNOS production, which dampens OXPHOS in M1 macrophages, promotes their metabolic and phenotypic reprogramming into the M2 phenotype^12^, reinforcing the link between mitochondrial OXPHOS and M2 polarization. OXPHOS in M2 macrophages is also fueled by the uptake of fatty acids oxidized by FAO^17^. A functional and intact TCA cycle is crucial for supplying the ATP demands for the high glycosylation (uridine diphosphate *N*-acetylglucosamine, UDP-GlcNAc) levels of lectin and CD206 necessary for M2 macrophage function^12^. Glutamine metabolism drives M2 polarization at multiple levels. Glutaminolysis supplies α-KG, which is essential for M2 OXPHOS and FAO^18^. Glutamine-derived α-KG causes epigenetic reprogramming for H3K27 demethylation on the promoters of M2 marker genes; it favors PHD activity, thereby reducing the HIF1α level^19^. Glutamine is also a substrate for UDP-GlcNAc synthesis. In addition, altered arginine metabolism plays a key role in macrophage polarization states and is one of the first characteristics used to classify them.

## Role of macrophages in female reproduction

### Macrophages during the reproductive cycle

The human endometrium is a dynamic organ that undergoes menstrual cycles consisting of proliferation, differentiation (decidualization), tissue breakdown (menstruation) and repair over 400 times during a woman’s reproductive life. The dynamic nature of the endometrium depends primarily on cyclic fluctuations in the ovarian steroid hormones estrogen and progesterone. During the proliferative phase, estrogen promotes revascularization to drive endometrial proliferation. Following ovulation, progesterone stimulates endometrial remodeling and stromal decidualization in preparation for pregnancy. In the absence of pregnancy, regression of the corpus luteum causes a rapid decline in progesterone levels, ultimately triggering menstruation^20^. Menstruation results in rapid breakdown and shedding of the functionalis layer, a differentiated part of the endometrium^20^.

Inflammatory cells, including monocytes, macrophages and neutrophils, are key mediators of the continuous breakdown and repair of the endometrium^21^. Macrophages constitute 1–2% and 3–5% of all endometrial cells during the proliferative and secretory phases, respectively. The macrophage population dramatically increases, reaching a peak of 6–15% during the menstrual phase following progesterone withdrawal^22^. During the secretory phase, when macrophage abundance increases, the human endometrium increases the expression of various chemokines, such as C–C motif chemokine ligand 4 (CCL4), CCL7, CCL21 and CCL22 (ref. ^23^). Changes in chemokine expression may reflect transient fluctuations in the number of macrophages throughout the menstrual cycle. The temporal and spatial regulation of macrophage location and trafficking within the endometrium is closely tied to these cyclic hormonal changes. Macrophages congregate around the endometrial glands during the secretory phase and accumulate in perivascular regions close to the luminal epithelium before the onset of menstruation^24^. This strategic positioning may enable macrophages to respond rapidly to tissue breakdown and initiate repair processes as menstruation begins. However, whether macrophages are retained within the uterine cavity or replaced during the cycle remains unclear. To address this question, several studies have used multiple markers to characterize the phenotypes of endometrial macrophages. Notably, CD68^+^CD163^+^ macrophages secrete both pro- and anti-inflammatory factors, highlighting the heterogeneity within endometrial macrophage populations^25^. In addition, macrophages interact with uterine natural killer (NK) cells and T cells throughout the menstrual cycle, dynamically modulating local inflammation and tissue remodeling to maintain endometrial health^26,27^. During the late secretory phase and early menstruation, uterine NK cells release IFN-γ, which promotes the M1-like activation of macrophages, enhancing their ability to clear apoptotic cells and tissue debris^28^. Moreover, regulatory T cells suppress excessive inflammation by secreting IL-10 and TGF-β, which support the M2 polarization of macrophages^29^. This coordinated action between macrophages and other immune cells is essential for preventing prolonged inflammation and ensuring effective tissue repair during the reproductive cycle.

### Macrophages during embryo implantation

Embryo implantation and parturition are biphasic proinflammatory events, whereas anti-inflammatory contexts predominate during the later stages of fetal development, suggesting that homeostatic immune balance in the endometrium is vital to ensure a successful pregnancy^30^. The mid-secretory phase (days 19–23 of the menstrual cycle) is called the ‘window of implantation’, when the uterus becomes receptive^24^. Macrophages with dendritic cells contribute to regulating the adaptive immune response as well as angiogenesis and tissue remodeling at the implantation site. Proinflammatory cytokines are crucial for embryo implantation and decidualization. For example, the levels of CSF-1, IL-6 and leukemia inhibitory factor (LIF) in the endometrium increase after embryo implantation^31^. CSF-1 promotes trophoblast growth, and CSF-1 null female mice exhibit infertile phenotypes^32^. Although LIF was first described as a cytokine that induces macrophage differentiation, it is indispensable for embryo implantation. Endometrial LIF deficiency causes complete failure of embryo implantation and decidualization in mice. LIF protein and mRNA levels in the human endometrium significantly increase during the window of implantation^33^. Furthermore, macrophage-derived cytokines promote the glycosylation of surface proteins in the luminal epithelium, which is required for blastocyst attachment^34^. For example, *Fut2*, a fucosyltransferase responsible for fucosylation, is upregulated in uterine epithelial cells in vitro following coculture with macrophages or LIF and IL-1β^35^. Accordingly, when uterine macrophages are diminished in vivo, uterine epithelial *Fut2* mRNA levels are reduced. *FUT1* transfection into human endometrial cells alters the glycosylation patterns of important factors associated with embryo attachment, including MUC1 and integrin α5β1 (ref. ^36^).

### Macrophages during pregnancy

**Fig. 4:**
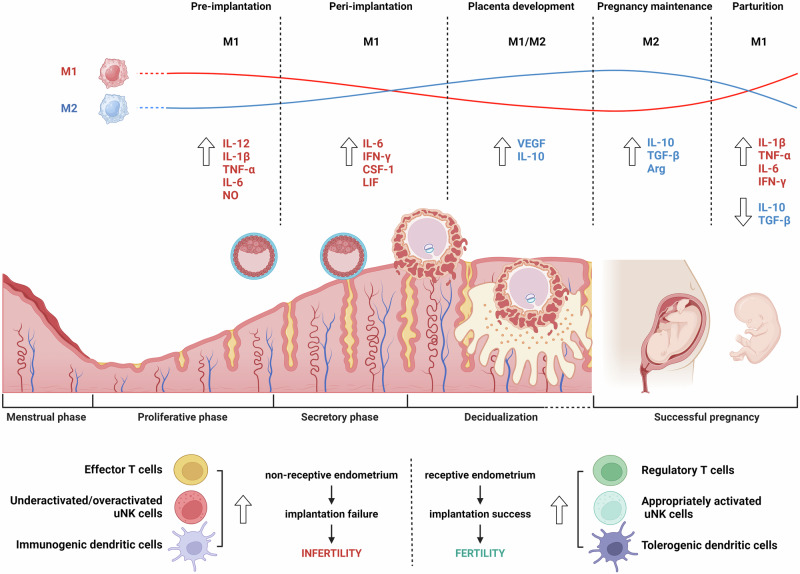
Immune modulation in the uterine microenvironment during the menstrual cycle and pregnancy. Dynamic changes in macrophage polarization and cytokine profiles throughout the menstrual cycle and pregnancy stages emphasize their roles in maintaining fertility and pregnancy. Notably, the balance between M1 and M2 macrophages shifts at each phase, with M1 macrophages predominating in inflammatory stages such as preimplantation and parturition, whereas M2 macrophages increase during placental development and pregnancy maintenance, promoting tissue repair and anti-inflammatory responses. The image was created with BioRender.com.

A successful pregnancy requires a unique immunological state, which simultaneously involves the immune tolerance of a semiallogeneic fetus and maintenance of the defense competence against pathogens for both the mother and the fetus^31,37^. Among immune cells at the maternal–fetal interface (MFI), macrophages represent approximately 20–30% of decidual immune cells, making them the second predominant leukocyte population after decidual NK cells^38^. Decidual macrophages and NK cells work closely together at the MFI to maintain immune homeostasis. Decidual macrophages secrete IL-15, which activates decidual NK cells to modulate immune responses and promote regulatory T cell differentiation, thereby suppressing maternal T cells and supporting fetal tolerance^39^. Throughout pregnancy, macrophages remain highly abundant in gestational tissues and participate in tissue reconstruction and the rapid clearance of apoptotic cells to prevent aberrant immune activation against fetal alloantigens (Fig. 4)^40^.

During decidualization, a prerequisite for successful pregnancy, stromal cells undergo dramatic morphological changes from elongated fibroblasts to rounded epithelial-like cells that secrete various growth factors and cytokines for tissue remodeling^40^. Progesterone promotes endometrial leukocyte trafficking and proliferation in situ during the secretory phase, but the absence of progesterone receptors in endometrial macrophages precludes its direct regulation^41^. However, progesterone increases the expression of chemoattractants that are predominantly secreted by decidualizing cells^42^. For example, the increase in CCL4 during decidualization, followed by its subsequent decline, may contribute to the observed changes in macrophage abundance during this process^43^.

Decidual macrophages exhibit unique phenotypes and affect pivotal events throughout pregnancy, including trophoblast invasion, spiral arterial remodeling, tissue reconstruction and maternal–fetal tolerance^38,44^. Recent studies have suggested that decidual macrophages cannot be classified simply as M1 or M2 macrophages. Decidual macrophages are skewed toward the M1 phenotype during the peri-implantation period and then transition to a profile of mixed M1/M2 macrophages when extravillous trophoblasts invade the uterine stroma. This mixed population remains until an adequate placental–fetal blood supply is established. Subsequently, decidual macrophages shift toward the M2 phenotype to prevent rejection of the semiallogenic fetus. CD11c^high^ and CD11c^low^ macrophages, two different subsets of CD14^+^ decidual macrophages in the first trimester, show distinct expression profiles associated with tissue remodeling, growth and development^45^. These two populations secrete both proinflammatory and anti-inflammatory cytokines that may cooperatively contribute to the balance that establishes fetal–maternal tolerance, suggesting that they do not meet the conventional M1–M2 dichotomy classification. Decidual macrophage dysfunction in terms of number and M1 deviation may lead to adverse pregnancy outcomes, such as preterm labor, spontaneous miscarriage, preeclampsia and fetal growth restriction^14^. For example, the number of M1 macrophages around the spiral arteries increases in pregnancies complicated by preeclampsia, whereas trophoblast cell invasion simultaneously decreases.

## Macrophages in female reproductive diseases

The activation status of macrophages fluctuates regularly during different phases of the menstrual cycle in healthy women, as described in the section 'Macrophages during the reproductive cycle. Changes in the abundance and phenotypes of endometrial macrophages have been reported in several reproductive disorders. Elucidating the role of endometrial macrophages in these conditions may provide insights into disease pathogenesis and help to identify novel therapeutic targets^24^.

### PCOS

Polycystic ovary syndrome (PCOS) is one of the most common endocrine and metabolic disorders in reproductive-aged women. PCOS is characterized by numerous small cysts in the ovary, and its pathological conditions are characterized mainly by chronic anovulation, hyperandrogenism and/or infertility^46^. This disorder may be accompanied by aberrant regulation of lipid and glucose metabolism, frequently leading to insulin resistance and hyperinsulinemia^47,48^. Chronic ovarian inflammation (low-grade systemic inflammation) is a critical pathological feature of PCOS. The activation states of macrophages in peripheral blood exhibit periodic changes during the reproductive cycle^49^, suggesting that macrophage polarity is influenced by steroidal sex hormones. In the peripheral blood and ovaries of patients with PCOS during the follicular phase, the number of M1 macrophages increases with increasing M1:M2 ratio, accompanied by elevated levels of proinflammatory cytokines, such as IL-6, IL-18, TNF and CCL4 (ref. ^50^). While the number of CD163^+^ M2 macrophages is significantly greater in the endometrial tissue of patients with PCOS (PCOSE) than in that of healthy women during the mid-secretory phase, the level of TNF is also increased^51^. Given that adverse pregnancy outcomes such as miscarriage, pregnancy-induced hypertension and preterm labor are associated with PCOS, it is likely that the PCOS phenotype may affect normal endometrial function, promoting pregnancy complications^50^. This disturbed condition can affect glucose uptake in the endometrium and cause fertility failure in these women^52^. The increase in CD163^+^ M2 macrophages in PCOSE may be a feedback event that counteracts hyper-proinflammatory reactions in PCOSE^51^. However, the underlying mechanism by which abnormal endometrial macrophages affect fertility in women with PCOS still needs to be explored.

### RIF/RSA

Repeated implantation failure (RIF) is determined when good-quality embryos fail to implant following several in vitro fertilization cycles^53^. Macrophages are associated with trophoblast invasion and tissue and blood vessel remodeling during early pregnancy. Increased miR-153-3p expression in the decidual macrophages of patients with RIF attenuates trophoblast cell proliferation and migration. Macrophage depletion can lead to implantation failure in the mouse uterus^54,55^. Thus, abnormal macrophage distribution, number and polarization may be associated with RIF^56^. CD163^+^ decidual macrophages increase significantly in number and accumulate in the endometrial glands of patients with RIF^30^. The levels of CCL2, which regulates monocyte/macrophage migration via CCR2, increase in decidual cells during early pregnancy. CCL2 levels are higher in patients with RIF than in healthy women. Blocking The CCL2‒CCR2 axis increases M1-related HIF1α in macrophages^6^, suggesting that increased CCL2 in patients with RIF may overpolarize toward the M2 phenotype. Unlike those in patients with RIF, decidual macrophages in patients with unexplained recurrent spontaneous abortion (RSA) exhibit a shift toward M1 polarization. Reduced miR-103 promotes STAT1/IRF1 signaling, leading to M1 polarization in patients with RSA^57^. miR-146a-5p enhances embryo survival by promoting decidual macrophage polarization toward the M2 phenotype but is reduced in patients with RSA with increased M1 polarization^58^. Imbalanced macrophage polarization may disrupt and promote proinflammatory microenvironments at the MFI in early pregnancy, leading to failure of embryo implantation and pregnancy maintenance; however, the underlying mechanisms have not been clarified.

### AS

Asherman’s syndrome (AS) is characterized by intrauterine adhesion (IUA) with fibrosis, leading to symptoms such as hypomenorrhea, infertility and recurrent miscarriage^59^. Low-dose aspirin treatment or estrogen therapy has been applied to ameliorate AS phenotypes, but the endometrial tissues of patients with severe AS show inefficiently recovery^60^. Operative procedures can induce physical trauma to the endometrium, especially the basal layer, where endometrial stem cells are presumably present, often leading to IUA. Moreover, infections can cause chronic endometrial inflammation, which induces IUA^61^. In patients with AS, fibrotic tissue replaces the endometrial stroma and reduces cell proliferation. Hamilton proposed a balance between CSF-1- and GM-CSF-mediated anti- and proinflammatory activities during tissue injury and repair^62^. When the ratio is tipped toward GM-CSF, a proinflammatory response dominates until the inflammatory stimulus diminishes, after which the balance shifts to a CSF-1-mediated reparative/homeostatic state. Reduced CSF-1 levels in patients with AS may be responsible for the reduced number of M2 macrophages. Thus, manipulating macrophage phenotypes and functions may provide a therapeutic tool for treating inflammatory disorders such as AS^63–65^.

Mesenchymal stem cells (MSCs) derived from various tissues effectively cross-talk with macrophages to modulate their functions and phenotypes during tissue regeneration. We previously demonstrated that MSC transplantation and MSC-derived cyclophilin A restore the impaired endometrium in a mouse model of human AS^66^. Cyclophilin A activates its receptor, CD147, on macrophages to promote M1-to-M2 conversion in vitro and in vivo to alleviate AS phenotypes (Song et al., unpublished observation). Pretreatment of MSCs with melatonin improved their beneficial effects on endometrial regeneration and pregnancy outcomes, such as increasing macrophage recruitment and M1-to-M2 conversion and reducing inflammation and fibrosis in a rat model of AS^64^. Human umbilical cord MSC-derived exosomes with collagen scaffolds significantly promoted M2 macrophage polarization with a reduced inflammatory response, leading to endometrial regeneration^63^. Exosomes derived from adipose MSCs after TNF-stimulated gene 6 overexpression mitigated IUA by reducing endometrial fibrosis in mice. TNF-stimulated gene 6-modified exosomes effectively inhibited the activation of inflammatory M1-like macrophages during the initial stages of inflammation and maintained balanced macrophage polarization during the repair phase^67^. We recently reported that miR-10a-encapsulated macrophage-targeting liposomes were able to rebalance M1-biased macrophage polarity to restore healthy uterine environments in mice with AS^65^.

### Endometriosis

**Fig. 5:**
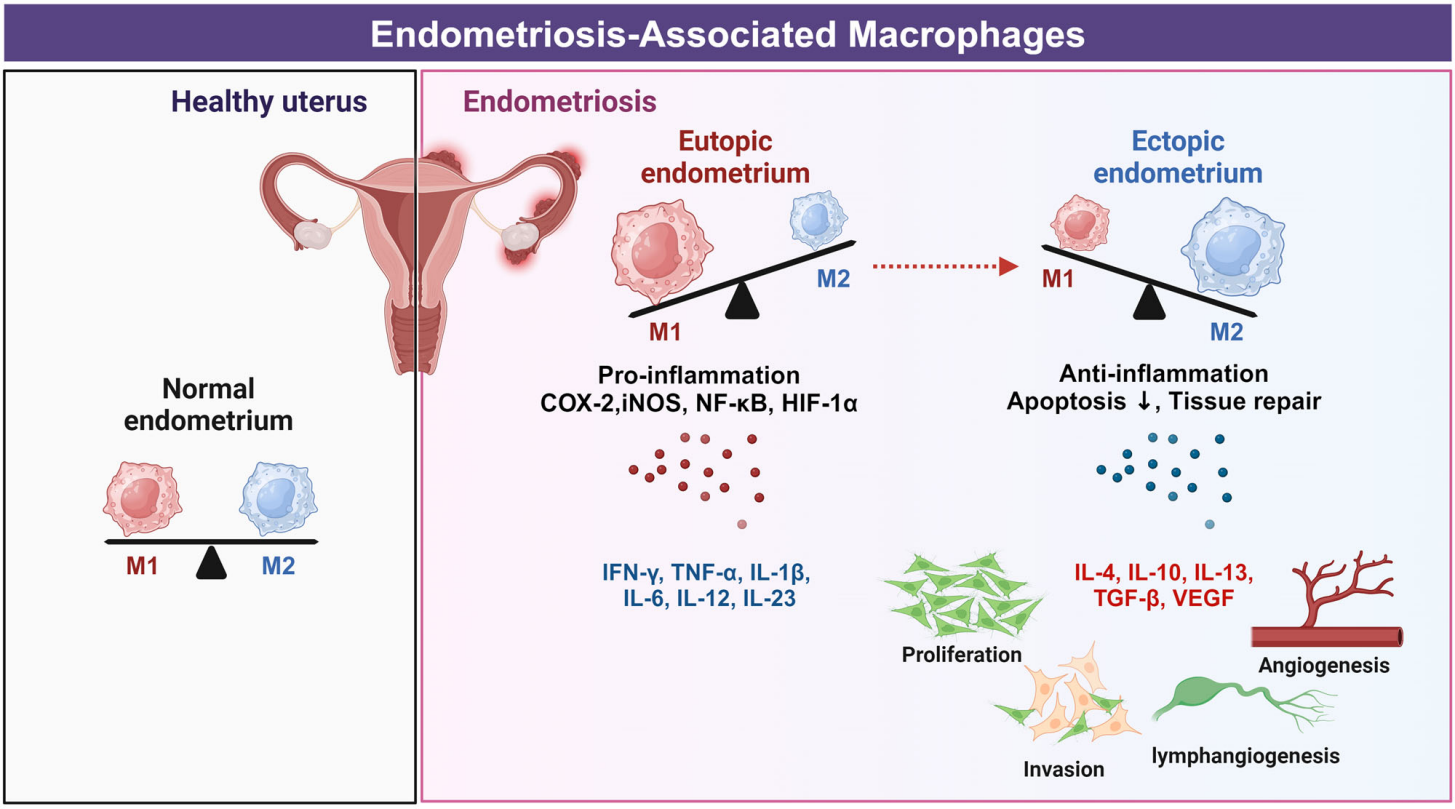
Imbalanced macrophage polarization is involved in the pathogenesis of endometriosis. Differential macrophage polarization and underlying molecular profiles in the healthy endometrium versus those in endometriosis. M1 and M2 macrophages are balanced in a healthy endometrium, maintaining normal endometrial function. In endometriosis, M1 macrophages predominate in the eutopic endometrium, leading to increased inflammation, whereas M2 macrophages in ectopic lesions promote tissue repair, angiogenesis and lesion progression. The image was created with BioRender.com.

Endometriosis is a common estrogen-dependent inflammatory disease that affects 10–15% of women of reproductive age worldwide, up to 50% of women with chronic pelvic pain and 30–50% of women with infertility^68^. The etiology of endometriosis is commonly retrograde menstruation, in which the shed endometrium flows backward into the fallopian tubes and peritoneal cavity, where it can adhere to and establish vascular networks and develop its nerve supply. Endometriosis is described as a ‘disease of the macrophage’^69^ (Fig. 5). In addition to increasing pain through nerve innervation, macrophages contribute to the growth and vascularization of endometriotic lesions. Women with endometriosis present increased macrophage numbers not only in the peritoneal fluid and ectopic lesions but also in the eutopic endometrium^70,71^.

Macrophages scavenge and phagocytose sloughed endometrial fragments during menstruation. These fragments are recognized by scavenger receptors, including CD36, whose expression is decreased in women with endometriosis. This defective scavenging ability may partly explain the persistence of endometriosis in these patients^24^. In the phagocytosis of aged or damaged erythrocytes, if Fe^2+^ clearance is inappropriately regulated, the accumulated Fe^2+^ is highly toxic, leading to apoptosis. Thus, excessive Fe^2+^ uptake can compromise macrophage function. Similarly, women with endometriosis present an increased abundance of Fe^2+^-overloaded macrophages in the peritoneal fluid^24^. Increased prostaglandin E_2_ inhibits the expression of annexin A2 and CD36 in peritoneal macrophages, weakening phagocytosis in patients with endometriosis and, thereby, contributing to its pathogenesis. Long noncoding RNAs, such as CHL1-AS1, in exosomes derived from peritoneal macrophages promote the proliferation, migration and invasion of ectopic stromal cells and inhibit their apoptosis^72^, suggesting that long noncoding RNAs participate in endometriosis pathogenesis. Tissue-resident macrophages have different origins that could dictate their function. M1 macrophages are enriched in the eutopic endometrium, whereas macrophages in the ectopic endometrium (endometriotic lesions) are polarized toward the M2 type^73^. Macrophages in endometriotic lesions can be derived from the endometrium, peritoneal cavity and/or BM. Endometriosis triggers the continuous recruitment of monocytes that differentiate into macrophages, which differ ontogenically from those within the peritoneal cavity. By depleting different populations, Hogg et al. found evidence suggesting that endometrial macrophages are ‘pro-endometriosis’, whereas monocyte-derived peritoneal macrophages are ‘anti-endometriosis’, acting to protect the cavity from lesion establishment^74^. Depletion of CD206^+^ M2 macrophages significantly decreases the formation of endometriotic-like lesions in mice. M2 macrophages promote the progression of endometriosis by secreting key factors such as VEGF-A and TGF-β, which drive angiogenesis and provide a favorable environment for ectopic lesions. Moreover, STAT3 signaling is activated in M2 macrophages within endometriotic lesions, further promoting the survival and proliferation of ectopic endometrial cells^30,70^. For example, the number of M2 macrophages was observed to increase upon exposure to exosomes and homogenates from ectopic endometriotic lesions in vitro, implying that endometriosis is responsible for the reduced M1:M2 ratio in patients.

Macrophages may be involved in endometriosis-associated pain, which is a type of neuropathic pain. This is supported by the presence of macrophages and nerve fibers in close proximity within endometriotic lesions. Netrin-1 is an axon guidance cue that directs axonal attraction and rejection during neural injury and regeneration. The expression levels of Netrin-1 are significantly greater in the serum and endometriotic lesions of women with endometriosis and are positively correlated with the severity of endometriosis-associated pain. Notably, Netrin-1 is coexpressed with the CD68 macrophage marker in endometriotic lesions. CD68^+^ macrophages are concentrated at nerve bundle sites within peritoneal lesions in a mouse model of endometriosis^75^. The abundances of CD14^high^ and CD14^low^ peritoneal macrophages are negatively and positively correlated with endometriosis pain scores, respectively. The concentration of macrophage-derived IGF-1 is also elevated in the peritoneal fluid of patients with endometriosis, with a positive correlation between IGF-1 concentration and pain score, possibly because IGF-1 enhances nerve sensitization and neurogenesis^76^.

### PTB

Preterm birth (PTB) is a major determinant of neonatal morbidity and mortality. In 2020, 13.4 million newborns were estimated to be born preterm (<37 weeks) worldwide (9.9% of all births)^77^. Chronic respiratory illnesses, neurodevelopmental disorders and long-term cognitive impairment are among the problems that occur after PTB. Although various factors affect PTB, infection and inflammation are well-defined risk factors. Notably, in one study 79% of patients with extreme PTB tested positive for an infectious insult^78^. However, the underlying mechanisms leading to PTB/preterm labor are complex and poorly understood.

The number of decidual macrophages remains stable throughout gestation. In mice, a greater number of endometrial macrophages at 15 days postcoitus (4 days before birth) decreased to nonpregnant levels a day before birth. The number of M1 macrophages in the decidua of patients with spontaneous PTB is much greater than that in the decidua of women with term labor. This dynamic nature of macrophages is also observed in pregnant rats, where uterine NO levels are elevated during pregnancy but reduced during term labor. NO, which can be produced by macrophages, inhibits myometrial contractions. A timely decrease in the number of macrophages with reduced NO levels is required for appropriate labor onset^79^. The importance of the homeostatic function of macrophages during pregnancy and labor has been reinforced by various experiments. The genetic depletion of maternal CD11b^+^ myeloid cells leads to PTB and adverse neonatal outcomes in mice^80^. Depletion of the elevated proinflammatory mediator levels and leukocyte activation in the decidua and increased neutrophil levels were associated with contractility in the myometrium. We also reported that the pharmacological depletion of maternal macrophages via clodronate liposomes resulted in an 80% incidence of PTB (Song et al., unpublished observation). During inflammation-induced preterm labor in mice, the Notch pathway is activated in decidual macrophages, promoting M2-to-M1 conversion^81^. Macrophages are skewed toward M1-positive and M1/M2 double-positive cells in the uteri of patients with inflammation-induced PTB. Macrophage infiltration and M1-like macrophage polarization in the preterm placenta may trigger decidual activation for labor through the production of PGs and proinflammatory cytokines^82^. In contrast, the activation of PPARγ with rosiglitazone attenuates the macrophage-mediated proinflammatory response and prevents PTB in mice^82^. A model of intra-amniotic inflammation showed that adoptive transfer of in vitro M2-polarized macrophages reduces PTB and improves neonatal survival. M2-polarized macrophages reduce the levels of proinflammatory mediators in the maternal blood and amniotic cavity and downregulate inflammatory events in the fetal brain and lung^83^. As this field of research is still evolving, clarifying macrophage plasticity during pregnancy and term labor could significantly contribute to the development of strategies for pregnancy management and PTB prevention.

## Macrophage therapy for inflammatory diseases and cancers

Macrophage therapy is an emerging research area in regenerative medicine and immunotherapy^84^. Therapies that increase macrophage recruitment and modulate macrophage activation can accelerate the healing process under various conditions of tissue injury and inflammation^85^. Inflammatory diseases, such as rheumatoid arthritis (RA) and inflammatory bowel disease (IBD), are associated with dysregulated immune responses^86^. Various approaches have been explored to harness the potential of macrophages for therapeutic purposes in different diseases via animal models^6^, encouraging further human clinical trials. Vitamin D and roburic acid promote the M1-to-M2 conversion of macrophage polarity, reduce the inflammatory milieu and improve tissue repair in animal models of colitis and RA, respectively^86,87^. We observed that the adoptive transfer of BM-derived M2 macrophages rebalances macrophage polarity and rescues impaired phenotypes, including fibrosis, in the uteri of mice with AS (Song et al., unpublished observation). In mice with myocardial infarction, the intramyocardial transplantation of in vitro M-CSF- and IL-4-primed ‘reparative’ macrophages has shown beneficial effects over nonprimed M0 (non-activated macrophage) BMDMs (bone marrow-derived macrophages)^85^. In hepatic liver injury models, the adoptive transfer of M2-like BMDMs from mice and humans reduces liver injury, fibrosis and several inflammatory mediators^85^. These findings have paved the way for ongoing human clinical trials, bringing us closer to potential therapeutic applications.

The therapeutic application of anti-TNF agents, such as infliximab and adalimumab, has shown promising outcomes in some patients with IBD and RA. Infliximab increases the number of CD68^+^CD206^+^ macrophages in patients with IBD, a systemic disease with immune abnormalities, especially those with a relatively high M1:M2 ratio^86^. Adalimumab promotes an anti-inflammatory M2 phenotype in macrophages from patients with RA^88^. Although several macrophage-targeted therapies are already being evaluated in clinical trials, many challenges remain for their successful clinical translation. One of the primary challenges is the complexity of the macrophage activation state in human tissues, which can differ significantly from that observed in animal models^89^. This heterogeneity makes it difficult to predict the in vivo effects of macrophage-targeted therapies in patients. Achieving precise delivery to target tissues and maintaining the functional phenotype of therapeutic macrophages is another major limitation to be addressed in this field.

With advances in acellular therapy, the therapeutic potential of MSC-derived extracellular vesicles (MSC-EVs) has attracted increasing attention for the treatment of various inflammatory diseases in mice^90^. However, the therapeutic effects of MSC-EVs are nullified when macrophages are pharmacologically depleted^91^. MSC-EVs promote IL-10 expression in M2 macrophages^92^ and reduce the inflammatory response, resulting in decreased M1 marker expression and increased M2 marker expression^93^. Furthermore, specific miRNAs in MSC-EVs have been investigated as potential therapeutics. For example, miR-let7 and miR-223-3p from BM-MSC-EVs target proinflammatory NF-κB and STAT3, respectively, to enhance M2 macrophage polarization^94,95^. Recent studies have suggested that macrophage reprogramming in situ via EVs derived from M2 macrophages (M2-EVs) is a promising approach for treating inflammatory diseases. M2-EVs efficiently rebalance M1-biased macrophage polarization and reduce inflammatory responses for wound healing and/or damage prevention in various tissues^96,97^. M2-EVs may inherit the anti-inflammatory properties of the original M2 macrophages and promote the establishment of an anti-inflammatory microenvironment under several conditions, such as colitis and airway inflammation^98^. MSC-EVs and/or M2-EVs are, thus, promising therapeutic(s) for alleviating the inflammatory environment and phenotypes of various endometrial disorders, but they remain unexplored in this field. Despite their potential, scaling up the production of EVs for clinical use poses a significant challenge, as does ensuring batch-to-batch consistency^99^. Furthermore, the biodistribution and pharmacokinetics of EVs are not yet fully understood, complicating the optimization of dosing and administration routes for effective clinical outcomes.

Cancer immunotherapy is a promising approach for treating malignancies. Chimeric antigen receptor (CAR) therapy is a novel cancer immunotherapy approach that integrates CAR structure and immune cell functions. CAR T cells are effective in the treatment of hematological malignancies; however, their poor entry into solid tumors renders them unsuitable for T-cell-based therapies. Given the constant trafficking of mononuclear phagocytes into tumors and the phagocytosis and killing activity of macrophages^85,100^, macrophage-based therapies might overcome this limitation. Human CAR macrophages (CAR-MФs) armed with receptors recognizing various molecules, such as CD19, CD22, HER2 and CD514, have been developed to attack hematopoietic and solid tumors^101^. CAR-MФs mediate phagocytosis, stably express M1 phenotypes and traffic to primary and metastatic tumors^102^. CAR-MФs targeting HER2 improved tumor clearance in a xenograft model of ovarian cancer^103^. Clinical trials are currently underway or planned to assess the potential of CAR-MФs in different tumors^100,104^. As immunotherapy advances, macrophage-based approaches offer promise for treating many diseases without unwanted side effects; however, further research and clinical trials are essential to determine the precise therapeutic window and establish robust protocols for macrophage-based therapies in humans^104,105^.

## Macrophage-specific targeting with a DDS

With the development of nanotechnology, drug delivery systems (DDSs) have attracted attention for delivering therapeutic agent(s) to disease sites, resulting in improved therapeutic efficacy with minimized toxicity and reduced nonspecific distribution^106,107^. DDSs based on materials such as lipids and polymers have been introduced to target macrophages through different routes^108^. Liposomes, artificial vesicles that mimic the structure of cell membranes, can be used as effective DDSs. Phosphatidylserine liposomes induce local M1-to-M2 macrophage polarization during bone regeneration Folic acid-modified liposomes repolarize M1 macrophages to M2 macrophages and alleviate RA phenotypes. Folic acid-modified liposomes significantly alleviate inflammatory responses in the limbs and decrease the serum levels of TNF and IL-1β^109^. We demonstrated that size-controlled miRNA-encapsulated liposomes specifically target macrophages to promote M1-to-M2 conversion and alleviate inflammation in vivo without causing cytotoxicity^65^. Because of their unique core-shell structure, micelles prepared from amphiphilic block copolymers have become a research hotspot in DDSs. Cationic micelles show significantly increased uptake efficiency in RAW 264.7 cells, a mouse macrophage line. Similar to liposomes, micelles with incorporated phosphatidylserine show enhanced macrophage recognition. Polymer nanoparticles (NPs), which can encapsulate or adsorb active compounds, offer advantages such as easy manufacturing, surface functionalization, biodegradability and biocompatibility. Some NPs can modulate macrophage polarization, and their effects depend on various modifications and residues. Carboxyl- and amino-modified polystyrene NPs inhibit M2 polarization by downregulating CD200R, CD163 and IL-10 expression without affecting M1 markers. Similarly, hydrophilic polyurethane NPs can suppress M1 polarization by reducing the levels of inflammatory mediators, such as TNF and IL-1β. Some inorganic NPs can also reprogram macrophage polarization. Ferumoxytol, an iron oxide NP, calcium-based NPs and manganese dioxide NPs can polarize macrophages toward the M1 phenotype, thereby inhibiting tumor growth. While many DDSs have been employed to modulate macrophage polarization in the treatment of inflammatory diseases and cancers, their application to reproductive disorders is still in its early stages. For example, octahedral nanoceria NPs combined with resveratrol (CeO_2_@RSV) alleviated endocrine dysfunction, inflammation and ovarian injury in a mouse model of PCOS. CeO_2_@RSV modulates proinflammatory microenvironments, repolarizing M1 macrophages into the M2 phenotype to reduce inflammation and enhance granulosa cell function in the ovary^110^. Considering that macrophage infiltration and macrophage-derived mediators are involved in PCOS pathogenesis, these findings suggest that CeO_2_@RSV is a promising therapeutic strategy for addressing inflammation and immune imbalance in PCOS. Further research in reproductive medicine is needed to advance and validate DDS applications, expanding therapeutic strategies for reproductive disorders.

## Conclusion

**Fig. 6:**
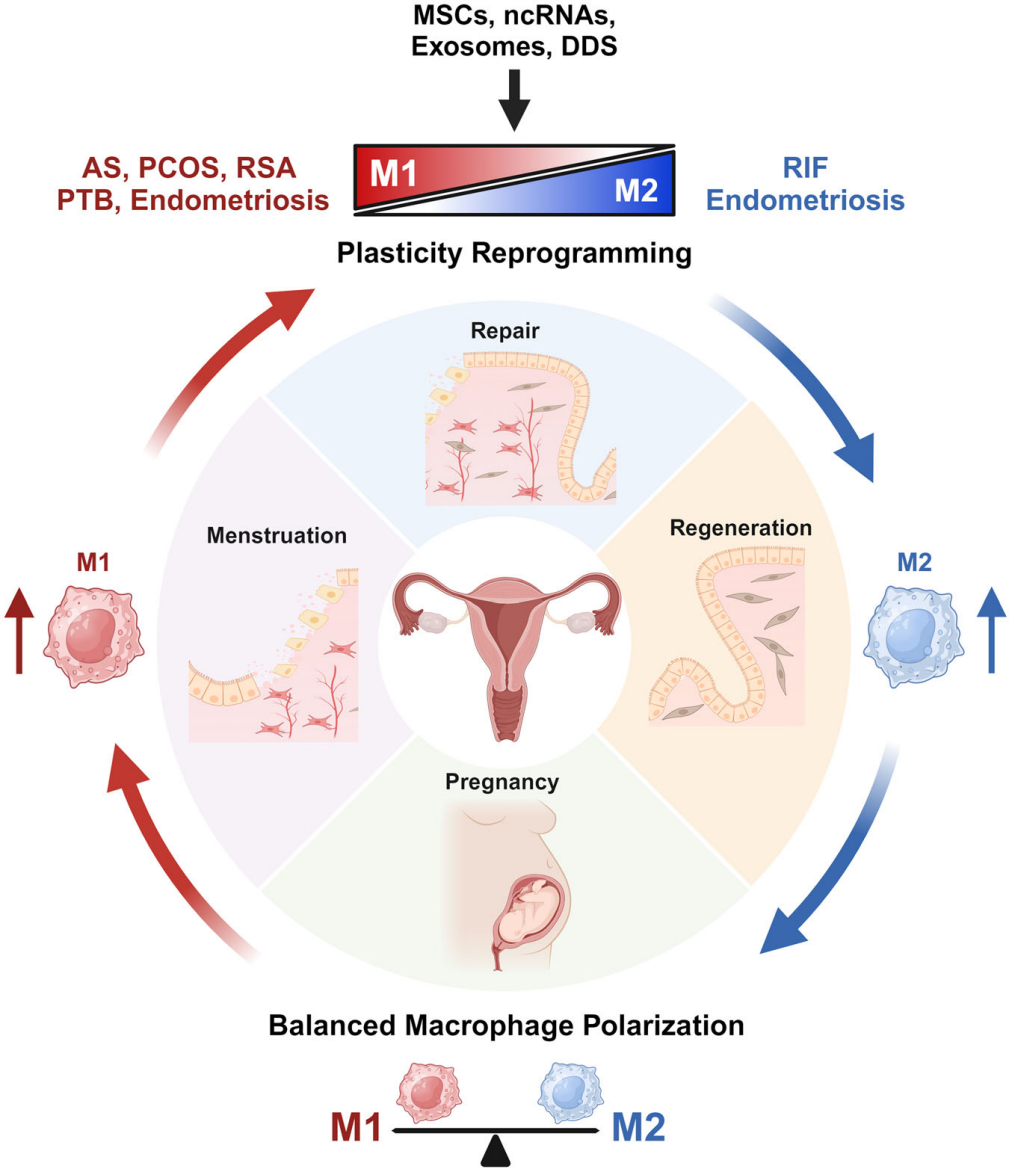
Macrophage polarization in uterine health and diseases. The importance of a homeostatic balance of macrophage polarization in uterine physiology and diseases and potential therapeutic strategies to modulate macrophage function for treating various uterine conditions. The image was created with BioRender.com.

Successful reproduction can be accomplished by fine tuning inflammation-like episodes and prevailing anti-inflammatory effects during other stages of pregnancy. Macrophages can acquire different phenotypes that frequently overlap in response to dynamic changes in the endometrial microenvironment, such as the menstrual cycle and pregnancy. Macrophages are not only simple bystanders that clear cell debris but also key players in orchestrating complex remodeling of the endometrium to maintain pregnancy. Aberrant phenotypes, numbers and locations of macrophages in the endometrium may affect normal physiology, underlying major events in female reproduction, which lead to diverse disorders associated with infertility. Although the design of therapeutic interventions to target macrophages has been challenging, several strategies involving phenotypic reprogramming and specific depletion are being pursued to recover balanced macrophage polarity. DDS-based nanomedicine using various biologics, such as MSCs, EVs and noncoding RNAs, has yielded promising outcomes in preclinical models, encouraging clinical trials. Advances in designing macrophage-specific targeting strategies with clinically proven DDSs, such as lipid NPs for mRNA delivery, are currently urged for clinical trials for reproductive disorders as well as other deadly diseases. Understanding how macrophages interact with other immune cells in the endometrium and how they influence reproductive outcomes would also provide valuable insights for developing novel macrophage-targeted therapies. Macrophage re-education is one of the most promising strategies for treating various cancers and inflammatory diseases, but it has just started in endometrial disorders and is aimed at the bidirectional reprogramming of macrophage plasticity between M1- and M2-like phenotypes (Fig. 6).
